# An open dataset about georeferenced harmonized national agricultural censuses and surveys of seven mediterranean countries

**DOI:** 10.1016/j.dib.2019.104774

**Published:** 2019-11-08

**Authors:** Ricardo Villani, Tiziana Sabbatini, Olga Moreno Perez, Nuno Guiomar, Marta Debolini

**Affiliations:** a Institute of Life Sciences, Sant’Anna School of Advanced Studies, Pisa, Italy; b Department of Economics and Social Sciences, Universitat Politècnica de València, Valencia, Spain; c ICAAM - Instituto de Ciencias Agrarias e Ambientais Mediterranicas, Universidade de Evora, Portugal; d UMR 1114 INRA-UAPV EMMAH, Avignon, France

**Keywords:** Mediterranean farming systems, Agricultural dynamics, Land systems, Crop diversity, West mediterranean basin, Crop production

## Abstract

The dataset presented in this paper is based on data gathered from several countries within the West Mediterranean area at the highest detailed scale regarding official statistics, with the aim of investigating land and food systems dynamics in the Mediterranean. Characterizing land and food systems dynamics is critical to reveal insights regarding interactions between current dynamics of agricultural practices, species diversity and local food systems. These interactions were analyzed, at multiple spatial scales, on a large part of the Mediterranean basin within the DIVERCROP Project (https://divercropblog.wordpress.com/).

An harmonized dataset with the desired characteristics was not readily available from official sources and, therefore, it was necessary to build an *ad hoc* database that could: (1) cover the Mediterranean areas of seven countries, namely Algeria (DZ), France (FR), Italy (IT), Malta (MT), Portugal (PT), Spain (ES) and Tunisia (TN); (2) contain data referred to the most disaggregated level of administrative units for which data is available in each country; (3) contain data referred to at least two time points, including the latest available data, in each country; (4) contain data on number of farm holdings, on the physical areas covered by the main annual and permanent crops and on livestock (number of heads); (5) contain a primary key that allows joining the census and surveys database to a geographical dataset of administrative units covering the entire area; (6) have an associated complete geographical dataset of administrative units, to allow spatial data analyses.

**Table:** Specifications Table

Subject area	Agriculture, Landscape Agronomy, Food security, Agricultural Economics
More specific subject area	Crop Production, Crop diversity, Food systems, Agricultural structure
Type of data	PostgreSQL Database, CSV File, shapefile
How data was acquired	Online acquisition of agricultural censuses, collection and standardization through collaboration with local partners. In particular, for the European Mediterranean countries and for Algeria, census data are nationally acquired through individual questionnaires to each farmer on the study area. For Tunisia, data are acquired through survey. The links to the national statistical services, where questionnaires and surveys are detailed, are listed on Appendix A.
Data format	Raw and partially elaborated data
Experimental factors	We describe the processing methods applied for building this harmonized and homogeneous dataset. We also show a simple example of possible application of the dataset for analysing agricultural dynamics.
Experimental features	We fully describe all the dataset variables and we give the access of the open dataset.
Data source location	Whole countries (Algeria, Italy, Malta, Portugal, Spain, Tunisia) and Mediterranean area of France
Data accessibility	Data provided in the article is accessible to the public at this link: https://divercropblog.wordpress.com/maps-data/

**Table:** 

**Value of the Data** • The data contain information referred to seven (European and African) Mediterranean countries, regarding number of holdings, cultivated areas and livestock. • The data was referred to at least two time points, allowing to analyze the evolution of the variables over time. • The data is spatially explicit, allowing to perform spatial analyses of the variables included in the dataset. • The data can reveal insights in terms of land and food systems dynamics over a large part of the Mediterranean basin.

## 1 Data

The dataset was originated from national agricultural censuses, where available, regarding the following countries: Algeria (DZ), France (FR), Italy (IT), Malta (MT), Portugal (PT), Spain (ES) and Tunisia (TN). It contains raw and partially elaborated data. The datafiles are available at this link: http://w3.avignon.inra.fr/gn_plateau_ressources/divercrop/. The database was built on PostgeSQL, an open source object-relational database management system. It stored data of the Mediterranean areas of seven European and African countries, regarding number of holdings, cultivated areas and livestock. The resulting dataset was made ready for linking to an *ad hoc* constructed shapefile, containing the same levels of detail of administrative units for which census and survey data were available, covering the entire area of interest.

Data from the national agricultural censuses (France, Italy, Malta, Portugal and Spain) and from agricultural surveys (Algeria and Tunisia) were collected for several time points. To standardize administrative units within the area of interest of the analysis, the European geocode standard for referencing the subdivisions of countries for statistical purposes was used, introducing adaptations of this standard where necessary (Algeria, Tunisia).

**Fig. 1:**
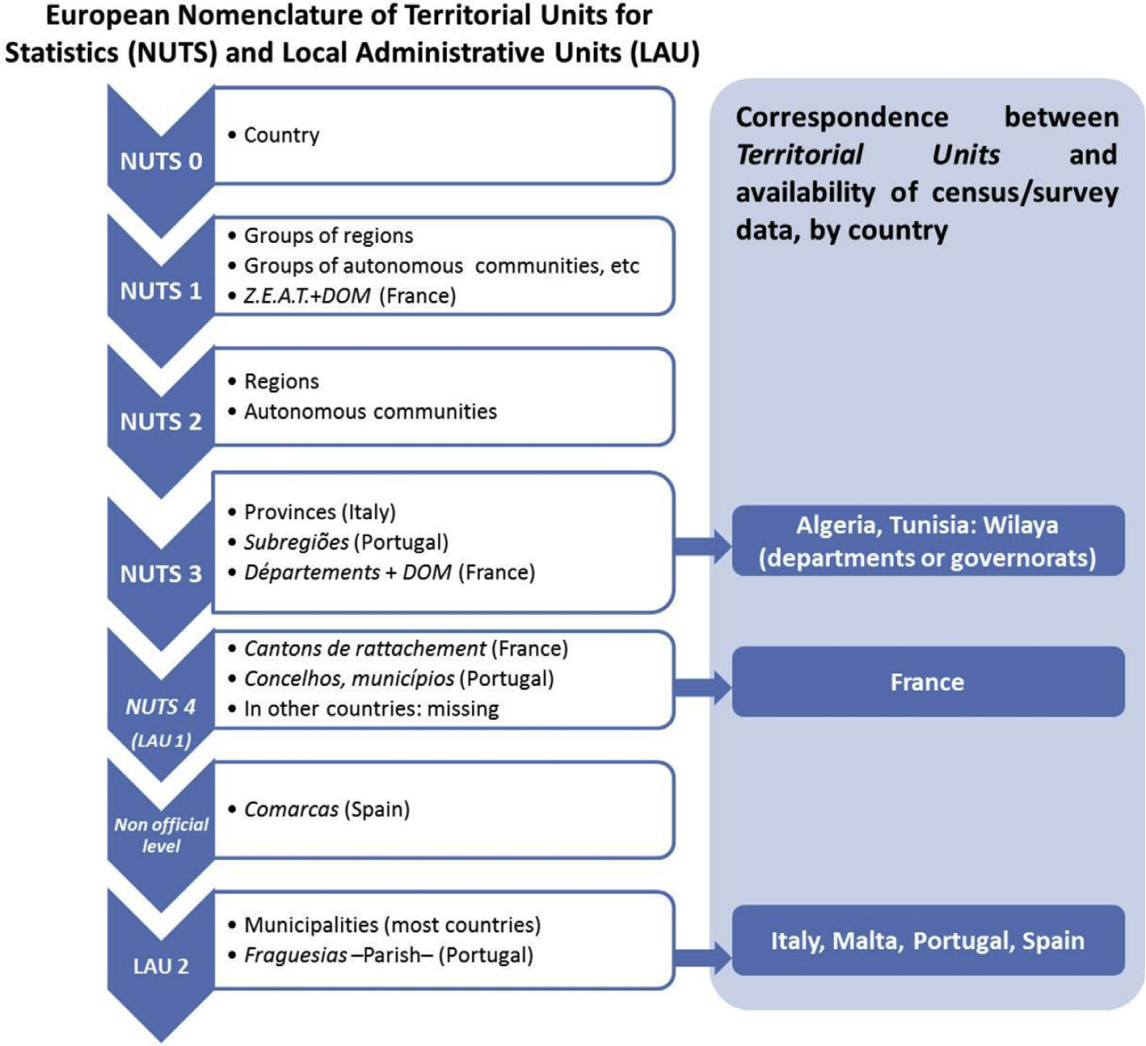
Hierarchy of the European Nomenclature of Territorial Units (left) and correspondence between the Territorial Unit levels and data availability, by country (right).

The spatial resolution and the correspondence of the territorial units of the different countries are showed on Fig. 1. Table 1 and Table 2 show respectively the primary key and keys for aggregation of data, and the years for which national agricultural datasets were incorporated into the database and the identifiers for those years. Table 3 describe most of the variables of the Agricultural Censuses Database, whereas the complete list of these variables is listed on Appendix 2.

**Table 1:** Primary key and keys for aggregation of data in the harmonized agricultural censuses database, corresponding to different levels of the hierarchy of administrative units.

Country	Level 1: **country**	Level 2: **nuts2**	Level 3: **nuts3** (including equivalent Wilaya)	Level 4: **lau1**	Level 5: **nuts3_lau2** (LAU 2)	Primary key: ***join_field*** (showing number of administrative units)
DZ-Algeria	X	N.A.	X	–	–	48
ES-Spain	X	X	X	–	X	8096
FR-France	X	X	–	X	–	1163
IT-Italy	X	X	X	–	X	8092
MT-Malta	X	N.A.	N.A.	–	X	68
PT-Portugal	X	X	X	–	X	4077
TN-Tunisia	X	N.A.	X	–	–	24
*Total number of administrative units*						*21,568*

In **bold**: names of the aggregator fields in the database. In the gray boxes: most disaggregated levels of the administrative units for which data were available.

**Table 2:** Years for which national agricultural datasets were elaborated. Column headings indicate the year codification in the database (y0 through y4).

	y0	y1	y2	y3	y4
DZ	–	–	_	2012	2016
ES	–	–	1999	2009	–
FR	–	–	2000	2010	–
IT	1982	1990	2000	2010	–
MT	–	–	2001	2010	–
PT	–	1989	1999	2009	–
TN	–	1995	2005	–	–

**Table 3:**
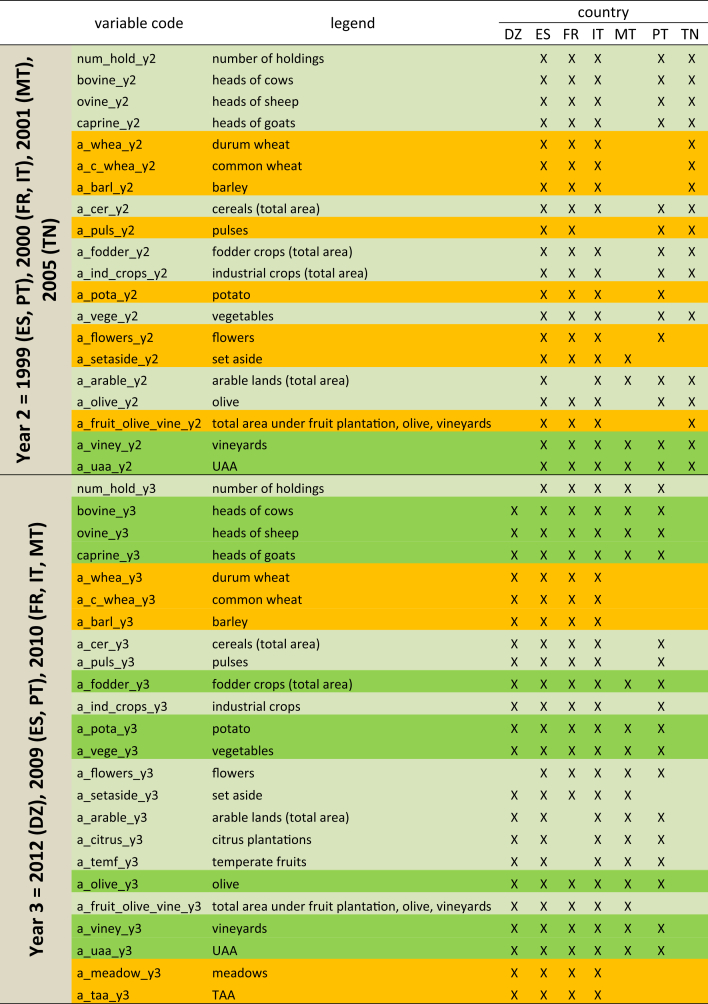
Variables of the Agricultural Censuses Database available for most countries. Backgrounds show: data available for 4 countries (orange), 5 countries (light green), 6 countries (dark green).

## 2 Experimental design, materials and methods

A harmonized national agricultural censuses and surveys database was built in order to gather the most relevant information from the censuses and surveys. During a first screening, all official data sources of national statistics, referred to each country involved in the DIVERCROP project, were consulted. In particular, the online databases of FAOSTAT and EUROSTAT were examined, along with the official web sites of national authorities for statistics. In Appendix 1, the complete list of links to these web resources is presented. Nevertheless, data could not be collected directly from Eurostat since the level of detail needed – the highest level of disaggregation of administrative units – was unavailable within the Eurostat databases. Indeed, Eurostat stores data aggregated at NUTS 2 level or higher, which was insufficient for the purpose of the characterization of land and food systems for which data was collected. As for the two North African countries, a centralized source of data was not available, thus for both European and African countries it was necessary to gather data directly from each country's individual official sources. In the specific cases of Tunisia, agricultural data sources were agricultural surveys, instead of censuses.

In most countries, data referred to the highest level of disaggregation of administrative units, which corresponds to the municipality or equivalent. In fact, data of 4 out of 7 countries, namely, Italy (municipality), Malta (cities), Portugal (parishes) and Spain (municipalities) referred to the most disaggregated administrative level, which is defined by the European geocode standard as *Local Administrative Unit Level 2* (*LAU 2*)^1^ while data referred to France needed aggregation at canton level, and the information provided by the two African countries, contained in agricultural surveys, was available at a higher level of geographical-administrative aggregation, namely *Wilaya* or governorats, as shown in Fig. 1. The lack of detailed data in these official sources led to the need for gathering the data by establishing a team of local contacts in each country, which provided raw and partially elaborated data. Gathering data from each country involved a serious drawback, since the codifications of the administrative units differed greatly from country to country. In fact, data gathered from each country were referred to administrative units that were codified following independent criteria, which implied the need to substitute the identification codes of the administrative units in each country with a harmonized codification system, which could be matched with a georeferenced dataset of administrative units.

### 2.1 Structure of the harmonized Agricultural Censuses Database

Table 1 shows the hierarchy of the administrative units, used as keys for aggregation in the relational database. Censuses data often referred to the local territorial units as they were in the year of the last agricultural census. In some cases, changes in the administrative units between agricultural censuses (i.e. between two censuses the territory corresponding to two or more municipalities may have been merged together) implied the use of individual georeferenced databases of administrative units for each country, as they were in the year when the census was held. To preserve the whole time-series without interruption in any single territorial unit, it was also necessary to have all local territorial units matching throughout the time series, which implied working manually on a case-by-case basis, particularly where merging areas of two or more territorial units or splitting of territorial units caused code changes over time. This task was carried out through direct support from the local contacts in each country.

Due to the inevitable diversity of sources of the geographical datasets, it was necessary to align all projections and to merge all datasets to obtain a unique coverage, which allows joining alphanumeric data from the Agricultural Censuses Database to the geographical dataset. The 3035 ETRS-LAEA European projection was used for this purpose. There was a shortcoming derived from this process of map composition that involved the geometry. In particular, the geographical dataset contains areas, at the international borders, that slightly overlap in some cases. In others, blank areas up to a few meters wide were generated when merging the individual national databases. Nevertheless, this drawback did not interfere with the purposes of the database and the quality of the analyses performed, since the integrity of the census data and of the location of each spatial unit was not affected by these slight inaccuracies of the geometry at the international borders.

The result of the work presented here was a georeferenced harmonized agricultural censuses and surveys database, composed of (1) a PostgreSQL database and (2) a shapefile with a common field allowing to represent the data on a map.

Table 2 shows, in relation with each country, the years for which national agricultural datasets were incorporated into the database and the identifiers for those years.

In Table 3, variables were classified according to the number of countries for which they were available in a given time point. Only variables available for at least 4 countries are shown, while Annex 2 shows the complete set of variables, with indication of their availability per country and per year.

In Table 3, values available in 4 countries are highlighted in orange, those available in 5 countries are shown with a light-green background, while the background is dark green when variables are available for 6 countries.

### 2.2 Descriptive statistics

**Fig. 2:**
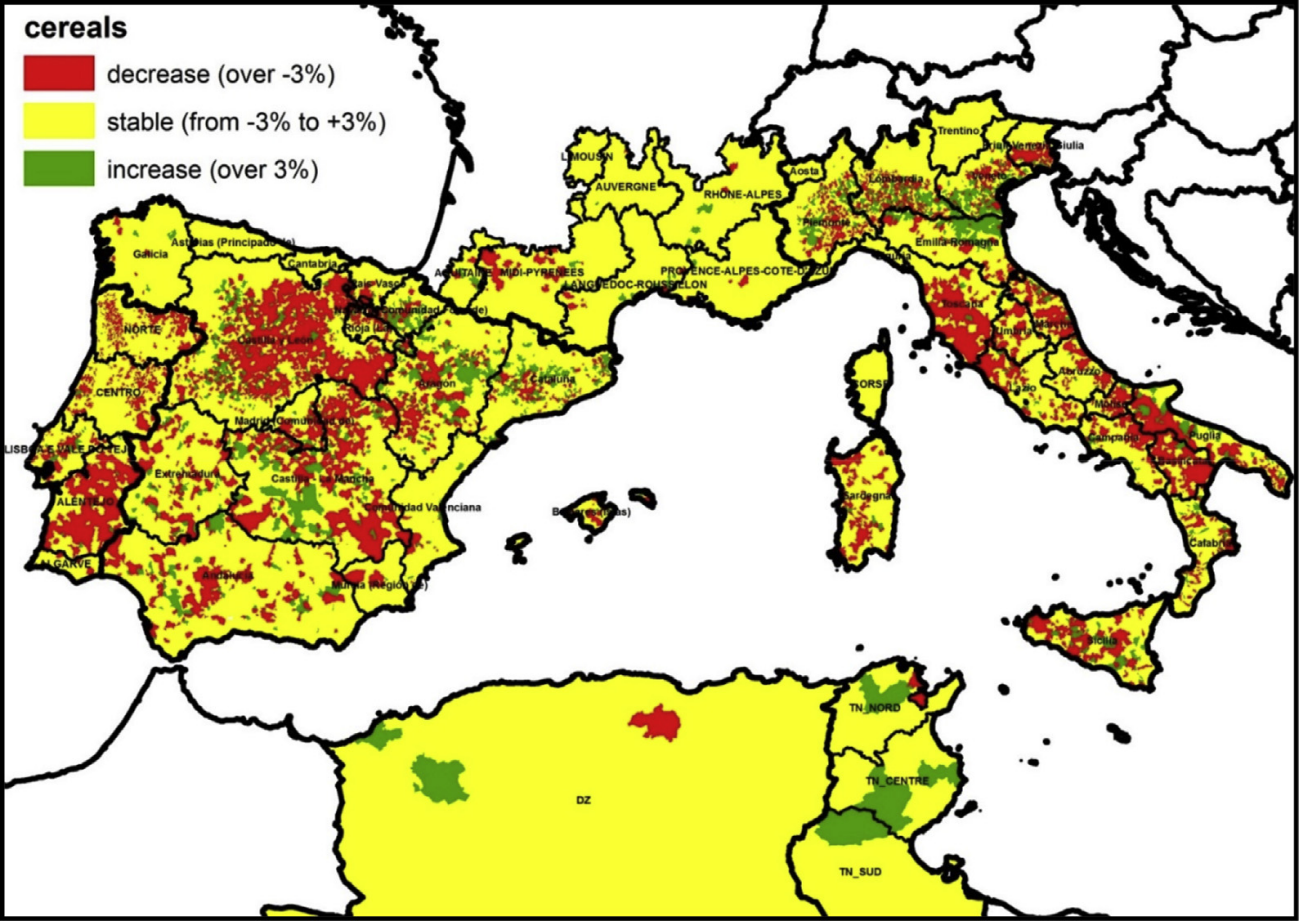
Map of the evolution of within a 10-year period of areas cultivated with cereal crops within the seven countries cover by the dataset.

As an example of the possible use of the database, in Fig. 2 we show a map representing the evolution of areas cultivated with cereal crops. Data are represented at the most disaggregated level of the administrative units for the area of interest, and it shows the evolution of areas cultivated with cereal crops within a 10-year period. When areas decreased by more than 3%, these were considered to have shown a process of decrement, when they changed within a range of −3% and 3%, they were considered stable, while if increment of cultivated areas was above 3% an increment was registered.

It can be observed that in most areas, surfaces dedicated to cereals cultivation were stable; this is particularly the case of most of France and the two African countries, and of large areas of Spain, mostly close to the coasts. Decrease in the areas under cereal cultivation were widespread in Italy, Portugal and Spain, particularly in the Italian regions of Tuscany, Marche, Molise, Puglia and Basilicata, in the Portuguese region of Alentejo and in the Spanish regions of Castilla y León and Castilla La Mancha. Finally, only some relatively restricted areas showed increment. This is mostly the case of the Italian regions of Emilia Romagna and Veneto and of almost all central regions of Spain, particularly Castilla La Mancha and Aragón.

All in all, the database has the potentiality of producing descriptive statistics at the most disaggregated level of administrative units over a large part of the Mediterranean area, including number of holdings, livestock and physical areas cultivated with the most widespread crops and categories of crops of the area, and to elaborate data regarding the evolution of these variables over time. These kind of analysis could improve the knowledge on land system dynamics at the overall Mediterranean scale, which are currently lacking [1,2]. Only by constructing a transnational database, in contrast with the single data sets obtained from national parties, it was possible to obtain a harmonized dataset that could allow the analysis of the agricultural dynamics over areas larger than that of a single country.

## Footnotes

1 To meet the demand for statistics at a local level, Eurostat maintains a system of Local Administrative Units (LAUs) compatible with NUTS. These LAUs are the building blocks of the NUTS, and comprise the municipalities and communes of the European Union (http://ec.europa.eu/eurostat/web/nuts/local-administrative-units).
