# Supplementary material for: An open dataset about georeferenced harmonized national agricultural censuses and surveys of seven mediterranean countries
Source: Data Brief. 2019 Nov 8;27:104774. doi: 10.1016/j.dib.2019.104774 (PMC6880016; doi:10.1016/j.dib.2019.104774)
Supplement: Multimedia component 1 [file mmc1.docx]

**Appendix 1**

**List of the national statistics authorities of DIVERCROP Countries**

**DZ-Algeria**

<http://www.ons.dz/-Donnees-Statistiques-.html>

<http://www.ons.dz/-Productions-vegetales-2005-2009,219-.html>

**ES-Spain**

<http://www.ine.es/dyngs/INEbase/es/operacion.htm?c=Estadistica_C&cid=1254736176851&menu=resultados&idp=1254735727106>

**FR-France**

<http://agreste.agriculture.gouv.fr/page-d-accueil/article/donnees-en-ligne>

**IT-Italy**

<http://dati.istat.it/?lang=en>

**MT-Malta**

no web resources available

**PT-Portugal**

<https://www.ine.pt/xportal/xmain?xpid=INE&xpgid=ine_bdc_tree&contexto=bd&selTab=tab2>

**TN-Tunisia**

<http://dataportal.ins.tn/fr/DataAnalysis?DRIlk3Kak0irBSijS5Jkg>
