# Supplementary material for: An open dataset about georeferenced harmonized national agricultural censuses and surveys of seven mediterranean countries
Source: Data Brief. 2019 Nov 8;27:104774. doi: 10.1016/j.dib.2019.104774 (PMC6880016; doi:10.1016/j.dib.2019.104774)
Supplement: Multimedia component 2 [file mmc2.docx]

**Appendix 2 – Complete details about dataset variables. Backgrounds show: data available for 4 countries (orange), 5 countries (light green), 6 countries (dark green). Backgrounds of the first column show: y0 (blue), y1 (violet), y2(light blue), y3 (brown), y4 (grey)**

|  |  |  | country | | | | | | |
| --- | --- | --- | --- | --- | --- | --- | --- | --- | --- |
|  | variable code | variable legend | DZ | ES | FR | IT | MT | PT | TN |
| Year 0 = 1982 (IT) | num_hold_y0 | number of holdings |  |  |  | X |  |  |  |
|  | bovine_y0 | heads of cows |  |  |  | X |  |  |  |
|  | ovine_y0 | heads of sheep |  |  |  | X |  |  |  |
|  | caprine_y0 | heads of goats |  |  |  | X |  |  |  |
|  | a_wood_plant_y0 | wood plantations |  |  |  | X |  |  |  |
|  | a_whea_y0 | durum wheat |  |  |  | X |  |  |  |
|  | a_c_whea_y0 | common wheat |  |  |  | X |  |  |  |
|  | a_barl_y0 | barley |  |  |  | X |  |  |  |
|  | a_rice_y0 | rice |  |  |  | X |  |  |  |
|  | a_maiz_r_y0 | maize, irrigated |  |  |  | X |  |  |  |
|  | a_maiz_y0 | maize |  |  |  | X |  |  |  |
|  | a_puls_y0 | pulses |  |  |  | X |  |  |  |
|  | a_cer_y0 | cereals (total area) |  |  |  | X |  |  |  |
|  | a_oil_y0 | annual oil crops (total area) |  |  |  | X |  |  |  |
|  | a_fodder_r_y0 | fodder crops, irrigated |  |  |  | X |  |  |  |
|  | a_fodder_y0 | fodder crops (total area) |  |  |  | X |  |  |  |
|  | a_toba_y0 | tobacco |  |  |  | X |  |  |  |
|  | a_sugb_y0 | sugarbeet |  |  |  | X |  |  |  |
|  | a_ind_crops_y0 | industrial crops (total area) |  |  |  | X |  |  |  |
|  | a_vege_r_y0 | vegetables, irrigated |  |  |  | X |  |  |  |
|  | a_vege_y0 | vegetables (total area) |  |  |  | X |  |  |  |
|  | a_flowers_y0 | flowers |  |  |  | X |  |  |  |
|  | a_setaside_y0 | set aside |  |  |  | X |  |  |  |
|  | a_arable_y0 | arable lands (total area) |  |  |  | X |  |  |  |
|  | a_citrus_r_y0 | citrus, irrigated |  |  |  | X |  |  |  |
|  | a_citrus_y0 | citrus plantations |  |  |  | X |  |  |  |
|  | a_temf_r_y0 | temperate fruits, irrigated |  |  |  | X |  |  |  |
|  | a_temf_y0 | temperate fruits (total area) |  |  |  | X |  |  |  |
|  | a_olive_y0 | olive |  |  |  | X |  |  |  |
|  | a_fruit_olive_vine_y0 | total area under fruit plantation, olive, vineyards |  |  |  | X |  |  |  |
|  | a_viney_r_y0 | vineyards, irrigated |  |  |  | X |  |  |  |
|  | a_viney_y0 | vineyards |  |  |  | X |  |  |  |
|  | a_uaa_y0 | UAA |  |  |  | X |  |  |  |
|  | a_meadow_y0 | meadows |  |  |  | X |  |  |  |
|  | a_uaa_r_y0 | UAA, irrigated |  |  |  | X |  |  |  |
|  | a_taa_y0 | TAA |  |  |  | X |  |  |  |
| Year 1 = 1990 (IT), 1989 (PT), 1995 (TN) | num_hold_y1 | number of holdings |  |  |  | X |  |  | X |
|  | bovine_y1 | heads of cows |  |  |  | X |  | X | X |
|  | ovine_y1 | heads of sheep |  |  |  | X |  | X | X |
|  | caprine_y1 | heads of goats |  |  |  | X |  | X | X |
|  | a_wood_plant_y1 | wood plantations |  |  |  | X |  |  |  |
|  | a_whea_r_y1 | durum wheat, irrigated |  |  |  |  |  |  | X |
|  | a_whea_y1 | durum wheat |  |  |  | X |  |  | X |
|  | a_c_whea_r_y1 | common wheat, irrigated |  |  |  |  |  |  | X |
|  | a_c_whea_y1 | common wheat |  |  |  | X |  |  | X |
|  | a_barl_y1 | barley |  |  |  | X |  |  | X |
|  | a_rice_y1 | rice |  |  |  | X |  |  |  |
|  | a_maiz_r_y1 | maize, irrigated |  |  |  | X |  |  |  |
|  | a_maiz_y1 | maize |  |  |  | X |  |  |  |
|  | a_ocer_r_y1 | other cereals, irrigated |  |  |  |  |  |  | X |
|  | a_ocer_y1 | other cereals |  |  |  |  |  |  | X |
|  | a_cer_r_y1 | cereals, irrigated |  |  |  |  |  |  | X |
|  | a_cer_y1 | cereals (total area) |  |  |  | X |  | X | X |
|  | a_chic_r_y1 | chickpea, irrigated |  |  |  |  |  |  | X |
|  | a_chic_y1 | chickpea |  |  |  |  |  |  | X |
|  | a_opul_r_y1 | other pulses, irrigated |  |  |  |  |  |  | X |
|  | a_opul_y1 | other pulses |  |  |  |  |  |  | X |
|  | a_puls_r_y1 | pulses, irrigated |  |  |  |  |  |  | X |
|  | a_puls_y1 | pulses |  |  |  | X |  | X | X |
|  | a_oil_y1 | annual oil crops (total area) |  |  |  | X |  |  |  |
|  | a_temp_grass_y1 | temporary grasses |  |  |  |  |  | X |  |
|  | a_fodder_r_y1 | fodder crops, irrigated |  |  |  | X |  |  |  |
|  | a_fodder_y1 | fodder crops (total area) |  |  |  | X |  | X | X |
|  | a_toba_y1 | tobacco |  |  |  | X |  |  |  |
|  | a_ind_crops_r_y1 | industrial crops, irrigated |  |  |  |  |  |  | X |
|  | a_ind_crops_y1 | industrial crops (total area) |  |  |  | X |  | X | X |
|  | a_pota_y1 | potato |  |  |  |  |  | X |  |
|  | a_sugb_y1 | sugarbeet |  |  |  | X |  |  |  |
|  | a_vege_r_y1 | vegetables, irrigated |  |  |  | X |  |  | X |
|  | a_vege_y1 | vegetables (total area) |  |  |  | X |  | X | X |
|  | a_flowers_y1 | flowers |  |  |  | X |  | X |  |
|  | a_other_arable_y1 | other arable crops |  |  |  |  |  | X |  |
|  | a_setaside_y1 | set aside |  |  |  | X |  |  |  |
|  | a_arable_y1 | arable lands (total area) |  |  |  | X |  | X | X |
|  | a_citrus_r_y1 | citrus, irrigated |  |  |  | X |  |  |  |
|  | a_citrus_y1 | citrus plantations |  |  |  | X |  | X |  |
|  | a_temf_r_y1 | temperate fruits, irrigated |  |  |  | X |  |  |  |
|  | a_temf_y1 | temperate fruits (total area) |  |  |  | X |  | X | X |
|  | a_nutf_y1 | nut fruits (total area) |  |  |  |  |  | X | X |
|  | a_table_olive_y1 | table olive |  |  |  |  |  |  | X |
|  | a_oil_olive_y1 | oil olive |  |  |  |  |  |  | X |
|  | a_olive_r_y1 | olive, irrigated |  |  |  |  |  |  |  |
|  | a_olive_y1 | olive |  |  |  | X |  | X | X |
|  | a_fruit_olive_vine_y1 | total area under fruit plantation, olive, vineyards |  |  |  | X |  |  | X |
|  | a_fruit_palm_y1 | fruit palms |  |  |  |  |  |  | X |
|  | a_permcrops_y1 | permanent crops |  |  |  |  |  | X |  |
|  | a_grape_y1 | grapes |  |  |  |  |  |  | X |
|  | a_viney_r_y1 | vineyards, irrigated |  |  |  | X |  |  |  |
|  | a_viney_y1 | vineyards |  |  |  | X |  | X | X |
|  | a_uaa_y1 | UAA |  |  |  | X |  | X | X |
|  | a_uaa_r_y1 | UAA, irrigated |  |  |  | X |  |  |  |
|  | a_meadow_y1 | meadows |  |  |  | X |  |  |  |
|  | a_taa_y1 | TAA |  |  |  | X |  |  |  |
| Year 2 = 1999 (ES, PT), 2000 (FR, IT), 2001 (MT), 2005 (TN) | num_hold_y2 | number of holdings |  | X | X | X |  |  | X |
|  | bovine_y2 | heads of cows |  | X | X | X |  | X | X |
|  | ovine_y2 | heads of sheep |  | X | X | X |  | X | X |
|  | caprine_y2 | heads of goats |  | X | X | X |  | X | X |
|  | a_wood_plant_y2 | wood plantations |  |  |  | X |  |  |  |
|  | a_whea_r_y2 | durum wheat, irrigated |  | X | X |  |  |  | X |
|  | a_whea_s_y2 | durum wheat, non irrigated |  | X |  |  |  |  |  |
|  | a_whea_y2 | durum wheat |  | X | X | X |  |  | X |
|  | a_c_whea_r_y2 | common wheat, irrigated |  | X | X |  |  |  | X |
|  | a_c_whea_s_y2 | common wheat, non irrigated |  | X |  |  |  |  |  |
|  | a_c_whea_y2 | common wheat |  | X | X | X |  |  | X |
|  | a_barl_r_y2 | barley, irrigated |  | X |  |  |  |  | X |
|  | a_barl_s_y2 | barley, non irrigated |  | X |  |  |  |  |  |
|  | a_barl_y2 | barley |  | X | X | X |  |  | X |
|  | a_oat_r_y2 | oat, irrigated |  | X |  |  |  |  |  |
|  | a_oat_s_y2 | oat, non irrigated |  | X |  |  |  |  |  |
|  | a_oat_y2 | oat |  | X | X |  |  |  |  |
|  | a_rye_r_y2 | rye, irrigated |  | X |  |  |  |  |  |
|  | a_rye_s_y2 | rye, non irrigated |  | X |  |  |  |  |  |
|  | a_rye_y2 | rye |  | X | X |  |  |  |  |
|  | a_rice_y2 | rice |  | X | X | X |  |  |  |
|  | a_maiz_r_y2 | maize, irrigated |  | X | X | X |  |  |  |
|  | a_maiz_s_y2 | maize, non irrigated |  | X |  |  |  |  |  |
|  | a_maiz_y2 | maize |  | X | X | X |  |  |  |
|  | a_ocer_r_y2 | other cereals, irrigated |  | X |  |  |  |  | X |
|  | a_ocer_s_y2 | other cereals, non irrigated |  | X |  |  |  |  |  |
|  | a_ocer_y2 | other cereals |  | X |  |  |  |  | X |
|  | a_cer_r_y2 | cereals, irrigated |  | X |  |  |  |  | X |
|  | a_cer_s_y2 | cereals, non irrigated |  | X |  |  |  |  |  |
|  | a_cer_y2 | cereals (total area) |  | X | X | X |  | X | X |
|  | a_chic_r_y2 | chickpea, irrigated |  | X |  |  |  |  | X |
|  | a_chic_s_y2 | chickpea, non irrigated |  | X |  |  |  |  |  |
|  | a_chic_y2 | chickpea |  | X |  |  |  |  | X |
|  | a_greenpea_r_y2 | greenpea, irrigated |  | X |  |  |  |  |  |
|  | a_greenpea_s_y2 | greenpea, non irrigated |  | X |  |  |  |  |  |
|  | a_greenpea_y2 | greenpea |  | X |  |  |  |  |  |
|  | a_opul_r_y2 | other pulses, irrigated |  | X |  |  |  |  | X |
|  | a_opul_s_y2 | other pulses, non irrigated |  | X |  |  |  |  |  |
|  | a_opul_y2 | other pulses |  | X |  |  |  |  |  |
|  | a_puls_r_y2 | pulses, irrigated |  | X |  |  |  |  | X |
|  | a_puls_s_y2 | pulses, non irrigated |  | X |  |  |  |  |  |
|  | a_puls_y2 | pulses |  | X | X | X |  | X | X |
|  | a_cott_r_y2 | cotton, irrigated |  | X |  |  |  |  |  |
|  | a_cott_s_y2 | cotton, non irrigatred |  | X |  |  |  |  |  |
|  | a_cotton_y2 | cotton |  | X |  |  |  |  |  |
|  | a_sunf_r_y2 | sunflower, irrigated |  | X | X |  |  |  |  |
|  | a_sunf_s_y2 | sunflower, non irrigated |  | X |  |  |  |  |  |
|  | a_sunf_y2 | sunflower |  | X | X |  |  |  |  |
|  | a_soyb_r_y2 | soybean, irrigated |  | X | X |  |  |  |  |
|  | a_soyb_s_y2 | soybean, non irrigated |  | X |  |  |  |  |  |
|  | a_soyb_y2 | soybean |  | X | X |  |  |  |  |
|  | a_rape_r_y2 | rapeseed, irrigated |  | X |  |  |  |  |  |
|  | a_rape_y2 | rapeseed |  | X | X |  |  |  |  |
|  | a_rape_s_y2 | rapeseed, non irrigated |  | X |  |  |  |  |  |
|  | a_oil_y2 | annual oil crops (total area) |  | X | X | X |  |  |  |
|  | a_perm_fodder_s_y2 | permanent fodder crops, non irrigated |  | X |  |  |  |  |  |
|  | a_perm_fodder_r_y2 | permanent fodder crops, irrigated |  | X |  |  |  |  |  |
|  | a_perm_fodder_y2 | permanent fodder crops |  | X |  |  |  |  |  |
|  | a_fodder_maiz_s_y2 | fodder maize, non irrigated |  | X |  |  |  |  |  |
|  | a_fodder_maiz_r_y2 | fodder maize, irrigated |  | X | X |  |  |  |  |
|  | a_fodder_maiz_y2 | fodder maize |  | X | X |  |  |  |  |
|  | a_fodder_legu_r_y2 | fodder legumes, irrigated |  | X |  |  |  |  |  |
|  | a_fodder_legu_s_y2 | fodder legumes, non irrigate |  | X |  |  |  |  |  |
|  | a_fodder_legu_y2 | fodder legumes |  | X |  |  |  |  |  |
|  | a_alfalfa_r_y2 | alfalfa, irrigated |  | X |  |  |  |  |  |
|  | a_alfalfa_s_y2 | alfalfa, non irrigated |  | X |  |  |  |  |  |
|  | a_alfalfa_y2 | alfalfa |  | X |  |  |  |  |  |
|  | a_temp_grass_y2 | temporary grasses |  |  |  |  |  | X |  |
|  | a_fodder_r_y2 | fodder crops, irrigated |  |  |  | X |  |  |  |
|  | a_fodder_y2 | fodder crops (total area) |  | X | X | X |  | X | X |
|  | a_toba_r_y2 | tobacco, irrigated |  | X |  |  |  |  |  |
|  | a_toba_s_y2 | tobacco, non irrigated |  | X |  |  |  |  |  |
|  | a_toba_y2 | tobacco |  | X |  | X |  |  |  |
|  | a_hop_r_y2 | hop, irrigated |  | X |  |  |  |  |  |
|  | a_hop_s_y2 | hop, non irrigated |  | X |  |  |  |  |  |
|  | a_hop_y2 | hop |  | X |  |  |  |  |  |
|  | a_arom_r_y2 | aromatic plants, irrigated |  | X |  |  |  |  |  |
|  | a_arom_s_y2 | aromatic plants, non irrigated |  | X |  |  |  |  |  |
|  | a_arom_y2 | aromatic plants |  | X | X |  |  |  |  |
|  | a_othind_r_y2 | other industrial crops, irrigated |  | X |  |  |  |  |  |
|  | a_othind_s_y2 | other industrial crops, non irrigated |  | X |  |  |  |  |  |
|  | a_othind_y2 | other industrial crops |  | X |  |  |  |  |  |
|  | a_ind_crops_s_y2 | industrial crops, non irrigated |  | X |  |  |  |  |  |
|  | a_ind_crops_r_y2 | industrial crops, irrigated |  | X |  |  |  |  | X |
|  | a_ind_crops_y2 | industrial crops (total area) |  | X | X | X |  | X | X |
|  | a_rts_r_y2 | roots, irrigated |  | X |  |  |  |  |  |
|  | a_rts_s_y2 | roots, non irrigated |  | X |  |  |  |  |  |
|  | a_rts_y2 | roots |  | X |  |  |  |  |  |
|  | a_pota_r_y2 | potato, irrigated |  | X | X |  |  |  |  |
|  | a_pota_s_y2 | potato, non irrigated |  | X |  |  |  |  |  |
|  | a_pota_y2 | potato |  | X | X | X |  | X |  |
|  | a_sugb_r_y2 | sugarbeet, irrigated |  | X | X |  |  |  |  |
|  | a_sugb_s_y2 | sugarbeet, non irrigated |  | X |  |  |  |  |  |
|  | a_sugb_y2 | sugarbeet |  | X | X | X |  | X |  |
|  | a_vege_r_y2 | vegetables, irrigated |  | X | X | X |  |  | X |
|  | a_vege_s_y2 | vegetables, non irrigated |  | X |  |  |  |  |  |
|  | a_vege_y2 | vegetables |  | X | X | X |  | X | X |
|  | a_flowers_y2 | flowers |  | X | X | X |  | X |  |
|  | a_other_arable_y2 | other arable crops |  |  |  |  |  | X |  |
|  | a_setaside_y2 | set aside |  | X | X | X | X |  |  |
|  | a_arable_y2 | arable lands (total area) |  | X |  | X | X | X | X |
|  | a_citrus_r_y2 | citrus, irrigated |  |  |  | X |  |  |  |
|  | a_citrus_y2 | citrus plantations |  | X |  | X |  | X |  |
|  | a_temf_r_y2 | temperate fruits, irrigated |  | X |  | X |  |  |  |
|  | a_temf_s_y2 | temperate fruits, non irrigated |  | X |  |  |  |  |  |
|  | a_temf_y2 | temperate fruits (total area) |  | X |  | X |  | X | X |
|  | a_nutf_r_y2 | nut fruits, irrigated |  | X |  | X |  |  |  |
|  | a_nutf_s_y2 | nut fruits, non irrigated |  | X |  |  |  |  |  |
|  | a_nutf_y2 | nut fruits (total area) |  | X |  |  |  | X | X |
|  | a_table_olive_r_y2 | table olive, irrigated |  | X |  |  |  |  |  |
|  | a_table_olive_s_y2 | table olive, non irrigated |  | X |  |  |  |  |  |
|  | a_table_olive_y2 | table olive |  | X |  |  |  |  | X |
|  | a_oil_olive_y2 | oil olive |  | X |  |  |  |  | X |
|  | a_olive_r_y2 | olive, irrigated |  | X |  |  |  |  |  |
|  | a_olive_s_y2 | olive, non irrigated |  | X |  |  |  |  |  |
|  | a_olive_y2 | olive |  | X | X | X |  | X | X |
|  | a_fruit_olive_vine_y2 | total area under fruit plantation, olive, vineyards |  | X | X | X |  |  | X |
|  | a_fruit_palm_y2 | fruit palms |  |  |  |  |  |  | X |
|  | a_permcrops_y2 | permanent crops |  | X |  |  |  | X |  |
|  | a_grape_s_y2 | grapes, non irrigated |  | X |  |  |  |  |  |
|  | a_grape_r_y2 | grapes, irrigated |  | X |  |  |  |  |  |
|  | a_grape_y2 | grapes |  | X |  |  |  |  | X |
|  | a_viney_r_y2 | vineyards, irrigated |  | X | X | X |  |  |  |
|  | a_viney_s_y2 | vineyards, non irrigated |  | X |  |  |  |  |  |
|  | a_viney_y2 | vineyards |  | X | X | X | X | X | X |
|  | a_uaa_y2 | UAA |  | X | X | X | X | X | X |
|  | a_meadow_y2 | meadows |  | X | X | X |  |  |  |
|  | a_uaa_r_y2 | UAA, irrigated |  |  |  | X |  |  |  |
|  | a_taa_y2 | TAA |  | X | X | X |  |  |  |
| Year 3 = 2012 (DZ), 2009 (ES, PT), 2010 (FR, IT, MT) | num_hold_y3 | number of holdings |  | X | X | X | X |  |  |
|  | bovine_y3 | heads of cows | X | X | X | X | X | X |  |
|  | ovine_y3 | heads of sheep | X | X | X | X | X | X |  |
|  | caprine_y3 | heads of goats | X | X | X | X | X | X |  |
|  | a_wood_plant_y3 | wood plantations |  |  |  | X |  |  |  |
|  | a_whea_r_y3 | durum wheat, irrigated |  | X | X |  |  |  |  |
|  | a_whea_s_y3 | durum wheat, non irrigated |  | X |  |  |  |  |  |
|  | a_whea_y3 | durum wheat | X | X | X | X |  |  |  |
|  | a_c_whea_r_y3 | common wheat, irrigated |  | X | X |  |  |  |  |
|  | a_c_whea_s_y3 | common wheat, non irrigated |  | X |  |  |  |  |  |
|  | a_c_whea_y3 | common wheat | X | X | X | X |  |  |  |
|  | a_barl_r_y3 | barley, irrigated |  | X | X |  |  |  |  |
|  | a_barl_s_y3 | barley, non irrigated |  | X |  |  |  |  |  |
|  | a_barl_y3 | barley | X | X | X | X |  |  |  |
|  | a_oat_r_y3 | oat, irrigated |  | X | X |  |  |  |  |
|  | a_oat_s_y3 | oat, non irrigated |  | X |  |  |  |  |  |
|  | a_oat_y3 | oat | X | X | X |  |  |  |  |
|  | a_rye_r_y3 | rye, irrigated |  | X |  |  |  |  |  |
|  | a_rye_s_y3 | rye, non irrigated |  | X |  |  |  |  |  |
|  | a_rye_y3 | rye |  | X | X |  |  |  |  |
|  | a_rice_y3 | rice |  | X | X | X |  |  |  |
|  | a_maiz_s_y3 | maize, non irrigated |  | X |  |  |  |  |  |
|  | a_maiz_r_y3 | maize, irrigated |  | X | X | X |  |  |  |
|  | a_maiz_y3 | maize |  | X | X | X |  |  |  |
|  | a_ocer_r_y3 | other cereals, irrigated |  | X |  |  |  |  |  |
|  | a_ocer_s_y3 | other cereals, non irrigated |  | X |  |  |  |  |  |
|  | a_ocer_y3 | other cereals |  | X |  |  |  |  |  |
|  | a_cer_y3 | cereals (total area) | X | X | X | X |  | X |  |
|  | a_chic_r_y3 | chickpea, irrigated |  | X |  |  |  |  |  |
|  | a_chic_s_y3 | chickpea, non irrigated |  | X |  |  |  |  |  |
|  | a_chic_y3 | chickpea | X | X |  |  |  |  |  |
|  | a_greenpea_r_y3 | greenpea, irrigated |  | X |  |  |  |  |  |
|  | a_greenpea_s_y3 | greenpea, non irrigated |  | X |  |  |  |  |  |
|  | a_greenpea_y3 | greenpea | X | X |  |  |  |  |  |
|  | a_opul_r_y3 | other pulses, irrigated |  | X |  |  |  |  |  |
|  | a_opul_s_y3 | other pulses, non irrigated |  | X |  |  |  |  |  |
|  | a_opul_y3 | other pulses | X | X |  |  |  |  |  |
|  | a_puls_y3 | pulses | X | X | X | X |  | X |  |
|  | a_cott_r_y3 | cotton, irrigated |  | X |  |  |  |  |  |
|  | a_cott_s_y3 | cotton, non irrigatred |  | X |  |  |  |  |  |
|  | a_cotton_y3 | cotton |  | X |  |  |  |  |  |
|  | a_sunf_r_y3 | sunflower, irrigated |  | X | X |  |  |  |  |
|  | a_sunf_s_y3 | sunflower, non irrigated |  | X |  |  |  |  |  |
|  | a_sunf_y3 | sunflower |  | X | X |  |  |  |  |
|  | a_soyb_r_y3 | soybean, irrigated |  | X | X |  |  |  |  |
|  | a_soyb_s_y3 | soybean, non irrigated |  | X |  |  |  |  |  |
|  | a_soyb_y3 | soybean |  | X | X |  |  |  |  |
|  | a_rape_r_y3 | rapeseed, irrigated |  | X | X |  |  |  |  |
|  | a_rape_s_y3 | rapeseed, non irrigated |  | X |  |  |  |  |  |
|  | a_rape_y3 | rapeseed |  | X | X |  |  |  |  |
|  | a_oil_y3 | annual oil crops (total area) |  | X | X | X |  |  |  |
|  | a_perm_fodder_r_y3 | permanent fodder crops, irrigated |  | X |  |  |  |  |  |
|  | a_perm_fodder_s_y3 | permanent fodder crops, non irrigated |  | X |  |  |  |  |  |
|  | a_perm_fodder_y3 | permanent fodder crops |  | X |  |  |  |  |  |
|  | a_fodder_maiz_s_y3 | fodder maize, non irrigated |  | X |  |  |  |  |  |
|  | a_fodder_maiz_r_y3 | fodder maize, irrigated |  | X | X |  |  |  |  |
|  | a_fodder_maiz_y3 | fodder maize |  | X | X |  |  |  |  |
|  | a_fodder_legu_s_y3 | fodder legumes, non irrigate |  | X |  |  |  |  |  |
|  | a_fodder_legu_r_y3 | fodder legumes, irrigated |  | X |  |  |  |  |  |
|  | a_fodder_legu_y3 | fodder legumes |  | X |  |  |  |  |  |
|  | a_temp_grass_y3 | temporary grasses |  |  |  |  |  | X |  |
|  | a_fodder_r_y3 | fodder crops, irrigated |  |  |  | X |  |  |  |
|  | a_fodder_y3 | fodder crops (total area) | X | X | X | X | X | X |  |
|  | a_toba_r_y3 | tobacco, irrigated |  | X |  |  |  |  |  |
|  | a_toba_s_y3 | tobacco, non irrigated |  | X |  |  |  |  |  |
|  | a_toba_y3 | tobacco |  | X |  | X |  |  |  |
|  | a_hop_r_y3 | hop, irrigated |  | X |  |  |  |  |  |
|  | a_hop_s_y3 | hop, non irrigated |  | X |  |  |  |  |  |
|  | a_hop_y3 | hop |  | X |  |  |  |  |  |
|  | a_arom_r_y3 | aromatic plants, irrigated |  | X | X |  |  |  |  |
|  | a_arom_s_y3 | aromatic plants, non irrigated |  | X |  |  |  |  |  |
|  | a_arom_y3 | aromatic plants |  | X | X |  |  |  |  |
|  | a_othind_r_y3 | other industrial crops, irrigated |  | X |  |  |  |  |  |
|  | a_othind_s_y3 | other industrial crops, non irrigated |  | X |  |  |  |  |  |
|  | a_othind_y3 | other industrial crops |  | X |  |  |  |  |  |
|  | a_ind_crops_r_y3 | industrial crops, irrigated | X |  |  |  |  |  |  |
|  | a_ind_crops_y3 | industrial crops | X | X | X | X |  | X |  |
|  | a_rts_r_y3 | roots, irrigated |  | X |  |  |  |  |  |
|  | a_rts_y3 | roots |  | X |  |  |  |  |  |
|  | a_rts_s_y3 | roots, non irrigated |  | X |  |  |  |  |  |
|  | a_pota_r_y3 | potato, irrigated |  | X | X |  |  |  |  |
|  | a_pota_s_y3 | potato, non irrigated |  | X |  |  |  |  |  |
|  | a_pota_y3 | potato | X | X | X | X | X | X |  |
|  | a_sugb_r_y3 | sugarbeet, irrigated |  | X | X |  |  |  |  |
|  | a_sugb_s_y3 | sugarbeet, non irrigated |  | X |  |  |  |  |  |
|  | a_sugb_y3 | sugarbeet |  | X | X | X |  |  |  |
|  | a_vege_r_y3 | vegetables, irrigated |  |  | X | X |  |  |  |
|  | a_vege_y3 | vegetables | X | X | X | X | X | X |  |
|  | a_flowers_y3 | flowers |  | X | X | X | X | X |  |
|  | a_other_arable_y3 | other arable crops |  |  |  |  |  | X |  |
|  | a_setaside_y3 | set aside | X | X | X | X | X |  |  |
|  | a_arable_y3 | arable lands (total area) | X | X |  | X | X | X |  |
|  | a_citrus_r_y2 | citrus, irrigated |  |  |  | X |  |  |  |
|  | a_citrus_y3 | citrus plantations | X | X |  | X | X | X |  |
|  | a_temf_r_y3 | temperate fruits, irrigated |  | X |  | X |  |  |  |
|  | a_temf_s_y3 | temperate fruits, non irrigated |  | X |  |  |  |  |  |
|  | a_temf_y3 | temperate fruits | X | X |  | X | X | X |  |
|  | a_trof_y3 | tropical fruits |  | X |  |  |  |  |  |
|  | a_nutf_r_y3 | nut fruits, irrigated |  | X |  |  |  |  |  |
|  | a_nutf_s_y3 | nut fruits, non irrigated |  | X |  |  |  |  |  |
|  | a_nutf_y3 | nut fruits |  | X |  |  |  | X |  |
|  | a_table_olive_s_y3 | table olive, non irrigated |  | X |  |  |  |  |  |
|  | a_table_olive_r_y3 | table olive, irrigated |  | X |  |  |  |  |  |
|  | a_table_olive_y3 | table olive |  | X |  |  |  |  |  |
|  | a_oil_olive_y3 | oil olive |  | X |  |  |  |  |  |
|  | a_olive_r_y3 | olive, irrigated |  | X | X |  |  |  |  |
|  | a_olive_s_y3 | olive, non irrigated |  | X |  |  |  |  |  |
|  | a_olive_y3 | olive | X | X | X | X | X | X |  |
|  | a_fruit_olive_vine_y3 | total area under fruit plantation, olive, vineyards | X | X | X | X | X |  |  |
|  | a_fruit_palm_y3 | fruit palms | X |  |  |  |  |  |  |
|  | a_permcrops_y3 | permanent crops |  | X |  |  | X | X |  |
|  | a_grape_s_y3 | grapes, non irrigated |  | X |  |  |  |  |  |
|  | a_grape_r_y3 | grapes, irrigated |  | X | X |  |  |  |  |
|  | a_grape_y3 | grapes | X | X | X |  |  |  |  |
|  | a_viney_r_y3 | vineyards, irrigated | X |  | X | X |  |  |  |
|  | a_viney_y3 | vineyards | X | X | X | X | X | X |  |
|  | a_uaa_y3 | UAA | X | X | X | X | X | X |  |
|  | a_meadow_y3 | meadows | X | X | X | X |  |  |  |
|  | a_uaa_r_y3 | UAA, irrigated |  |  | X | X |  |  |  |
|  | a_taa_y3 | TAA | X | X | X | X |  |  |  |
|  | a_kitchgard_y3 | kitchen gardens |  |  |  |  | X |  |  |
| Year 4 = 2016 (DZ) | bovine_y4 | heads of cows | X |  |  |  |  |  |  |
|  | ovine_y4 | heads of sheep | X |  |  |  |  |  |  |
|  | caprine_y4 | heads of goats | X |  |  |  |  |  |  |
|  | a_uaa_y4 | UAA | X |  |  |  |  |  |  |
|  | a_taa_y4 | TAA | X |  |  |  |  |  |  |
|  | a_arable_y4 | arable lands (total area) | X |  |  |  |  |  |  |
|  | a_setaside_y4 | set aside | X |  |  |  |  |  |  |
|  | a_whea_y4 | durum wheat | X |  |  |  |  |  |  |
|  | a_c_whea_y4 | common wheat | X |  |  |  |  |  |  |
|  | a_barl_y4 | barley | X |  |  |  |  |  |  |
|  | a_oat_y4 | oat | X |  |  |  |  |  |  |
|  | a_cer_y4 | cereals (total area) | X |  |  |  |  |  |  |
|  | a_fodder_y4 | fodder crops (total area) | X |  |  |  |  |  |  |
|  | a_meadow_y4 | meadows | X |  |  |  |  |  |  |
|  | a_ind_crops_y4 | industrial crops (total area) | X |  |  |  |  |  |  |
|  | a_vege_y4 | vegetables | X |  |  |  |  |  |  |
|  | a_greenpea_y4 | greenpea | X |  |  |  |  |  |  |
|  | a_opul_y4 | other pulses | X |  |  |  |  |  |  |
|  | a_chic_y4 | chickpea | X |  |  |  |  |  |  |
|  | a_puls_y4 | pulses | X |  |  |  |  |  |  |
|  | a_pota_y4 | potato | X |  |  |  |  |  |  |
|  | a_ind_crops_r_y4 | industrial crops | X |  |  |  |  |  |  |
|  | a_viney_r_y4 | vineyards, irrigated | X |  |  |  |  |  |  |
|  | a_olive_y4 | olive | X |  |  |  |  |  |  |
|  | a_fruit_palm_y4 | fruit palms | X |  |  |  |  |  |  |
|  | a_citrus_y4 | citrus plantations | X |  |  |  |  |  |  |
|  | a_temf_y4 | temperate fruits | X |  |  |  |  |  |  |
|  | a_viney_y4 | vineyards | X |  |  |  |  |  |  |
|  | a_grape_y4 | grapes | X |  |  |  |  |  |  |
|  | a_fruit_olive_vine_y4 | total area under fruit plantation, olive, vineyards | X |  |  |  |  |  |  |
